# Development of Smart Textile Materials with Shape Memory Alloys for Application in Protective Clothing

**DOI:** 10.3390/ma13030689

**Published:** 2020-02-04

**Authors:** Grażyna Bartkowiak, Anna Dąbrowska, Agnieszka Greszta

**Affiliations:** Department of Personal Protective Equipment, Central Institute for Labour Protection—National Research Institute, Wierzbowa 48 Str., 90-133 Lodz, Poland; andab@ciop.lodz.pl (A.D.); aggre@ciop.lodz.pl (A.G.)

**Keywords:** shape memory alloys (SMAs), smart material, smart protective clothing, thermal factors

## Abstract

The latest directions of research on the design of protective clothing concern the implementation of smart materials, in order to increase its protective performance. This paper presents results on the resistance to thermal factors such as flames, radiant heat, and molten metals, which were obtained for the developed smart textile material with shape memory alloys (SMAs). The laboratory tests performed indicated that the application of the designed SMA elements in the selected textile material system caused more than a twofold increase in the resistance to radiant heat (RHTI_24_ = 224 s) with an increase of thickness of 13 mm (sample located vertically with a load), while in the case of tests on the resistance to flames, it was equal to 41 mm (sample located vertically without a load) and in the case of tests on the resistance to molten metal, it was 17 mm (sample located horizontally).

## 1. Introduction

Significant progress in the field of materials engineering has enabled the construction of products that are more suited to the individual needs of the user and changeable environmental conditions. The working environment is a specific area of application of such products due to the presence of varied hazards to which a person can be exposed. Therefore, in order to ensure the safety of the worker, the use of protective clothing is often required.

The latest directions of research on the design of protective clothing concern the implementation of smart materials that receive stimuli directly from the human body and the environment, and then respond to them with significant physical, chemical, and biological changes, which are frequently reversible. A group of garments in which it is appropriate to use smart materials in order to improve their functionality is protective clothing against thermal factors (flames, radiant heat, and molten metal splash), especially when such factors occur in the form of thermal impulses [1]. 

The protective clothing currently used by workers is characterized by identical protective properties over the entire surface, which are adjusted to the maximum level of exposure to thermal factors. However, in terms of the risks associated with these factors, work in hazardous conditions is usually associated with periodic exposure to their impact in the form of thermal impulses, e.g., during control works. Thermal hazards are present in the metallurgical industry and steel mills, where mechanization of the production process has developed considerably in recent years, thus limiting the direct access of humans to high-risk zones. Therefore, smart protective clothing against thermal factors intended for such worksites should adjust its protective properties to the level of hazardous factors. Such clothing should immediately detect the presence of a thermal stimulus (thermal factor) from the environment and respond to it in the form of a reversible physical change, involving an increase in its thickness and a related increase in the resistance to heat. However, when a worker is not exposed to thermal factors, the clothing should be characterized by a minimal thickness. This will ensure a higher level of clothing ergonomics while the worker is outside the high-risk zone associated with the presence of thermal factors. 

Smart materials that have a great potential to achieve this goal are shape memory alloys (SMAs). These materials, under the influence of temperature stimuli, are capable of spontaneous and reversible changes of shape. This shape memory effect is due to thermoelastic martensitic transformation, which is accompanied by heat exchange. The transformation takes place between the austenite and martensite phases [2,3,4]. An important feature of SMAs that can be used in smart protective clothing is the two-way shape memory effect (TWSME), which results from appropriately conducted thermomechanical treatment (TT) [5,6,7,8,9]. The element made of SMA, having a particular shape in the high-temperature phase, after cooling to the temperature specific for the low-temperature phase of the alloy, adopts another given shape. This means that the element with TWSME is capable of taking two shapes: one at a high temperature and another at a low temperature. Moreover, the transition between both shapes is reversible [10]. The ability of SMAs to change their shape, size, or internal structure under the influence of a particular stimulus is the subject of intensive application research [11]. The works in this area are also associated with their applications in textiles and clothing [5,12,13,14,15,16,17,18,19,20,21,22,23,24]. For example, the active knits made of SMA wire have a great potential for use as innovative actuators, because these materials thanks to the hierarchically organized structure is able to produce complex three-dimensional actuation motions [25].

**Figure 1:**
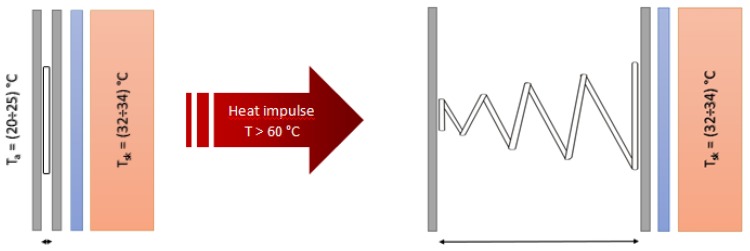
Idea of smart material with shape memory alloy (SMA) elements in the form of springs.

The concept of the application of SMAs in clothing structures for the smart adjustment of their thickness assumes that SMA springs be trained in such a way that they spontaneously increase their height under exposure to a thermal stimulus and reduce their height after its cessation (Figure 1).

As a consequence of temperature stimuli, an air gap is created between the fabrics and an increase in the resistance to heat transfer can be observed.

SMAs’ ability to change shape under specific conditions was used to develop smart protective clothing. Alloys with shape memory in the form of springs were introduced into textile materials for clothing protecting against thermal factors [14,26,27,28,29,30,31].

It has long been known that there is a close relationship between the size of air gaps in clothing and the thermal insulation of clothing. Song [32] conducted the flash fire manikin tests for the thermal protective coveralls, which showed that the burn predictions occur in the areas with the smaller insulation air gap. The numerical model developer by Song on the results of these tests predicts that the optimal size of the air gap between one-layer clothing and skin is 7–8 mm. A larger size of air gap may cause a heat transfer due to the natural convection and consequently increase the amount of total energy transferred to skin.

Wan and Stylios [31] showed that the performance of SMAs in cooperation with other materials applied in composites is an essential element in the design of active fabrics, taking into consideration the significant differences in the mechanical properties of these materials.

Research on the development of active textile systems with SMA elements for protection against the cold has been carried out by Yoo et al. [5], Pause [12], and Lee et al. [33]. Yoo et al. [5] incorporated SMA elements into clothing between the outer laminated fabric and lining. They proved that by using SMAs, it is possible to create a heat-insulating layer of air in clothing. At the same time, they stated that the developed clothing was not suitable due to the limited number of SMA locations introduced into the jacket. Pause [12] introduced the alloy with an activation temperature of approximately 5 °C between the two layers of nonwoven fabric. She used a wire that remained straight at a temperature above 5 °C. Below this temperature, it bent and repelled the outer nonwoven layer, causing an extension of the air gap between the nonwoven layers. The thickness of the thermal insulation layer increased from 20 to 24 mm, which resulted in an increase in the thermal resistance from 0.230 to 0.315 m^2^ × K/W. Lee et al. [33] developed a prototype mountain climbing jacket with 30 SMA springs that increased their height from 6 to 20 mm with a response temperature of 24.5 °C. The results of studies conducted with the participation of volunteers in a climatic chamber at 5 °C showed that the microclimate temperature under this clothing was higher than that of commercial clothing, and the subjective thermal, humidity, and comfort sensation were better.

The shape memory effect was also used to improve the protective properties against thermal factors in firefighting clothing [15,34,35,36]. White [15] implemented the pockets with rings made of SMAs between material layers in firefighter’s clothing to create expanding air spaces activated by heat. Laboratory tests showed that the use of the developed active elements improved the protective properties of clothing by 50% compared to firefighting clothing without SMAs. Park et al. [14] used the SMA elements in the form of springs. The tests also demonstrated an improvement in the protective properties of the developed clothing, as the time of effective protection increased by approximately 30% compared to standard firefighting clothing. Ma et al. [36] attached the SMA springs between the moisture barrier and thermal liner and investigated the temperature change at different layers under exposure to hot surface contact. It turned out that SMA springs could greatly decrease the heat transferred to the human body and improve thermal protection. In turn, Park et al. [37] assessed the impact of repeated laundering (50 cycles) on the SMA springs attached to the thermal liner and showed that silicon-attached springs remained intact after repeated laundering. He et al. [38] assessed the protective properties of multilayered systems for firefighters with SMA springs during a radiant heat exposure test (12 kW/m^2^) and hot surface contact test (400 °C). The results showed that the use of SMA springs improved the thermal protection of the system, but the extent to which the springs provided thermal protection was dependent on the arrangement mode and spring size. In turn, Lah et al. [39] proposed shape-memory nitinol knitted fabric with an austenite transition temperature of 75 °C for use in smart firefighting clothing to locally improve thermal insulation and protect the human skin from burns or overheating. This fabric was made of nickel titanium alloy monofils and achieved a two-way, shape memory effect by using a 15-cycle training process. The tests in a heated chamber at 100 °C showed that bulges measuring 12–25 mm in height were formed in the knitted fabric. 

Congalton [26] developed a bellows pocket model with the elements of SMAs in the form of conical springs for thermally activated protective clothing against heat and flames. This solution allowed an air gap 35 mm wide to be obtained between the layers of the pocket after activation at approximately 50 °C, which, in turn, resulted in increased local thermal insulation of the clothing. 

Russell et al. [40] achieved a similar effect by the attaching NiTi monofils in a grid pattern on a textile substrate and inserted this material between two fabric layers. The layer with NiTi wrinkles when the temperature of the environment rises to 70 °C and the air gap between fabric layers increase, thus increasing the thermal insulation of the whole system. There is known also a 3D NiTi knitted fabric based on a unique weft knitted pattern consisting of two layers that are periodically interlaced to form parallel hollow sections that reversibly deform in response to applied external loads and changing temperatures [41]. Another example is an intelligent textile material where SMA springs are attached between a cotton woven fabric and a thin Teflon foil. This material contracted when heated above 45 °C and expanded at lower temperatures [42]. When applied to the clothing, it could effectively develop a maximum air gap of 7.5 mm in a cold environment and a minimum air gap of 1.75 mm in a hot environment. 

The aforementioned solutions confirm the validity of the hypothesis that, by appropriate treatment of SMAs and their application between layers of protective clothing, it is possible to provide spontaneous adjustment of their properties to the level of risk in the work environment.

However, it is worth mentioning that depending on the application, the preparation of smart textile materials with SMA elements differs. This relates to both the selection and training of SMAs, as well as the selection of fabrics and integration method. Therefore, each application is unique and should be based on an analysis of the conditions at the particular work stand. 

The aim of this study is to develop and check the effectiveness of smart textile material with SMA elements for certain applications to clothing that protects against thermal hazards from work environments in the metallurgy industry. The novelty of this publication is the authorial program of the thermomechanical treatment of the NiTi shape memory alloy, the way SMA elements are implemented in the textile material, and the comprehensive testing program of smart materials with SMA elements, which is able to simulate real exposure to the main heat factors occurring in the work environment of steel workers (i.e., flames, radiant heat, and molten metal splashes).

## 2. Materials

### 2.1. Smart Elements with a Two-Way Shape Memory Effect

Taking into account the conditions of use of protective clothing, it was assumed that the expansion of textile materials’ system thickness and beginning of shape change of SMA elements should start at the moment when the outer surface of the textile system reaches a temperature above 60 °C, to ensure that the skin temperature remains below the pain threshold—that is, 44 °C [43]. Therefore, the martensitic transition of SMA elements for applications in clothing that protects against heat should occur in the temperature range from 50 to 60 °C [6].

In order to produce such smart elements intended for use in protective clothing, an NiTi shape memory alloy in the form of wire supplied by the Memry company (alloy M) after cold plastic processing was selected. It was characterized by a diameter equal to Ø 0.5 mm; chemical composition of Ni (55.6000%), Ti (44.3929%), Cr (0.0001%), Cu (0.0005%), and Fe (0.0065%); and the temperature of austenitic transformation (A_f_) of 55 ± 10 °C [6]. A smart element in the form of a cone-shaped spring was developed from this wire. Cone-shaped springs allow the minimum height of the smart element to be obtained in the low-temperature phase, which is equal to the thickness of the wire. On the contrary, in the case of cylindrical SMA elements, their height is equal to the wire thickness multiplied by the number of coils. As a consequence of the lowering of the height of the smart element in the low-temperature phase, there will also be a reduction in the distance between the protective clothing layers in the conditions of its common use. This will positively affect the ergonomic properties of clothing. Furthermore, when transmitting the spring of the conical shape in the low-temperature phase, it is possible to pull the spring axially in the opposite direction and thus force larger spring deformation than if it was only subjected to the maximum compression. 

**Figure 2:**
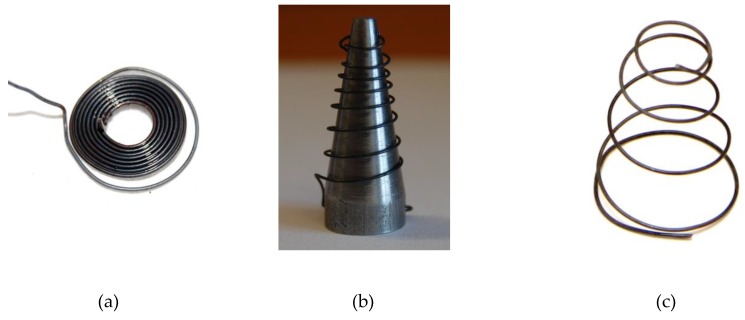
Views of smart elements during the thermomechanical treatment. (**a**) A view of the smart element obtained after the first heating step, (**b**) The spring put onto a metal cone for the second heating step, (**c**) A view of the element obtained after heating and cooling twice.

In order to obtain a cone-shaped spring with a repetitive shape change effect, a program for the thermomechanical treatment of SMA elements consisting of three stages was developed [44,45,46,47,48] as follows:

1. Wire was formed by winding it on a cylindrical mandrel. The first heating step was conducted at a temperature of 550 °C for 1 h, followed by cooling of the element in water at a temperature of approximately 5 °C. The flat spiral spring formed from the SMA wire was removed from the mandrel (Figure 2a), the spring was loosened and placed on a metal cone, and stable attachment of the ends of the wire to the cone was performed (Figure 2b);
2. The second heating step of the spring on the metal cone was pursued at a temperature of 550 °C for 1 h, and in order to obtain the shape memorized during the second heating step, rapid cooling of the element was conducted in water at a temperature of approximately 5 °C;
3. Heating of the decompressed spring to the temperature of approximately 70 °C (higher than the temperature of austenitic transformation) was conducted in order to obtain the shape memorized during the second heating step. Then, the deformed spring was cooled down in the air at a room temperature of approximately 20 °C, and it was compressed at that temperature. Axial dragging of the spring in the opposite direction to a length of about 8–10 cm was done in water at a temperature of 10 °C (above the temperature of martensitic transformation (M_f_)) and decompression of the spring in water occurred. The above steps were repeated 20 times. A view of the element obtained after heating and cooling twice is presented in Figure 2c.

**Figure 3:**
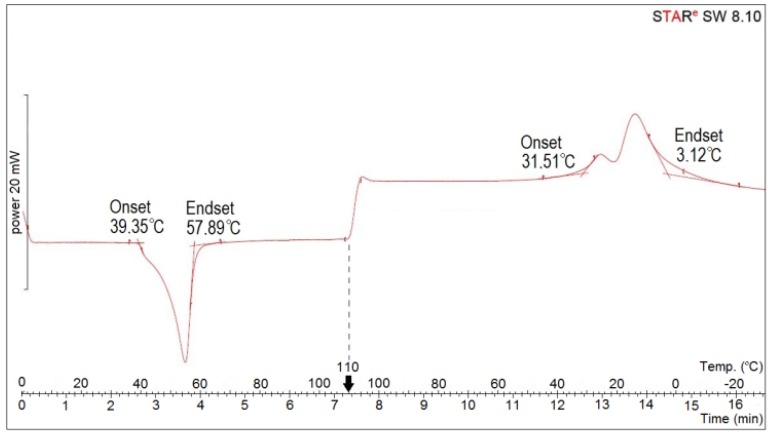
Differential scanning calorimetry (DSC) curve for SMA elements after thermomechanical cycling.

Smart elements in the form of cone-shaped springs of a 0.354–0.356 g weight had six coils each, the top diameter was 10 mm, and the bottom diameter was 20 mm. The height of the spring in the low-temperature phase was 0.5–2.0 mm, and in the high-temperature phase, it was 29.0–30.0 mm. To make each spring, approximately 265 mm of NiTi wire was used. Tests carried out using the differential scanning calorimetry method demonstrated that the shape change of the spring from the compressed to decompressed state occurred spontaneously in response to high temperature within the range of 39 to 58 °C, without applying any force, whereas the temperature of the change in the opposite direction ranged from 32 to 3 °C. The DSC (differential scanning calorimetry) curve for SMA elements after thermomechanical cycling is presented in Figure 3. This result is satisfactory and in compliance with the adopted assumption that the shape change of the active element should follow exposure to a thermal impulse at approximately 60 °C. The shape memory effect was checked for each produced element and found to be reproducible.

The results of spring deformation tests accompanying the shape change of the active element were evaluated during 50 cycles. Based on the deformation measurements occurring during the change of shape, it was found that deformation remains at about 79% after 50 cycles of work, and the variability of results in subsequent work cycles is below 5% [49]. This result indicates very good reproducibility of the SMA thermomechanical program and it was thus evaluated positively.

### 2.2. Smart Textile Materials with SMA Elements

In the next step, developed smart elements made of NiTi with TWSME were applied between two woven fabrics. For this purpose, fabrics characterized by flame-retardant properties and resistance to heat at a high temperature (180 °C), according to the current regulations for protective clothing against heat and flames (EN ISO 11612:2015) [50], were chosen. 

Preliminary studies have shown that the material set with aluminized woven fabric provides the best SMA springs expansion effect, without the material surface collapsing in the place where there are no SMA springs, so the fabric selected for the outer layer of smart textile material with SMAs was aluminized woven fabric (A) with para-aramid and oxidized poliacrylonitrile fibers, while the bottom layer was a lining fabric (P) made of 100% aramid fibers. The characteristics of the fabrics used in smart textile material containing SMAs are shown in Table 1.

**Figure 4:**
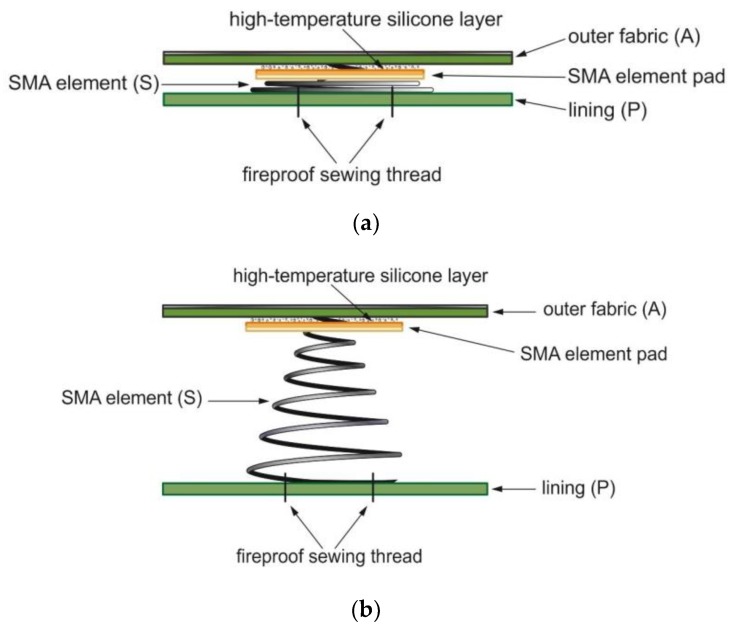
Design of smart textile material with SMA elements: (**a**) inactive state and (**b**) active state.

High-temperature silicone (ProSeal Clear RTV Silicone Adhesive and Sealant) and fireproof sewing thread made of 100% meta-aramid were applied for mounting the SMA elements on the fabrics. The bottom coil of each spring was stitched in two places to the lining fabric, whereas the upper one was mounted with high-temperature silicone on the fabric constituting of the outer layer of the active material through a pad in the form of nonwoven fabric laminated with a Teflon membrane, which formed a barrier to adhesive penetration. Such a method for the implementation of SMA elements for clothing ensures a high durability and aesthetics and prevents sticking of the spring coils. The developed smart textile material with SMA elements was marked with the symbol ASP. Figure 4a,b schematically present the protective material with SMA elements in an inactive and active state with visible SMA smart elements.

The principle of the smart textile material with SMA springs is based on the fact that active elements with SMA undergo spontaneous extension in the response of heat impulse (for example radiant heat). A distance in the form of an air layer is created between the layers of the system and, as a consequence, the system’s resistance to heat transfer increases. After the cessation of the heat factor (when the SMA temperature drops below 60 °C), the SMA elements shrink spontaneously, without applying any external force, causing the upper and lower layers of the system to slip (return to the initial state).

For comparative purposes, in order to determine how the extension of the SMA elements affects the improvement of the garment protective properties, outer fabric/lining material systems without SMA elements (marked with the symbol AP) were also prepared. Both variants of textile materials are presented in Table 2.

## 3. Testing Methods

In order to evaluate the performance of the developed smart textile material with SMA elements in terms of the resistance to thermal factors, laboratory tests according to the methods presented in Table 3 were carried out.

European standards were adopted as the basis for testing, according to which protective clothing used in the work environment is assessed. Testing methods have been modified to adapt them to active materials.

For each test, three samples of the above-mentioned materials were used.

### 3.1. Tests of Resistance to Ignition

The resistance to ignition of the developed textile materials was tested in accordance with EN ISO 15025:2016 [53]. The tests involved contact between an open flame and the surface of the test material, because, in this case, the flame is directed toward the smart SMA elements implemented in the material.

In accordance with this method, the surface of the material is exposed to a flame for 10 s, and the time of subsequent burning and incandescence is then measured. In addition, the flame spread (reaching the top or one of the vertical edges of the sample) is noted, as well as the formation of flaming debris or holes. In the case of smart textile materials with SMA elements, the increase in the sample thickness was additionally measured. The increase in the sample thickness was measured once after flame operation for three samples

### 3.2. Tests of Resistance to Radiant Heat

In order to determine the resistance to radiant heat of textile material (without SMAs), the materials were subjected to tests in accordance with a test method based on EN ISO 6942:2002 [54], and they were then assessed in accordance with the requirements of the EN ISO 11612:2015 standard [50] (Table 4).

Samples of the test materials were subjected to radiant heat at a heat flux density of Q*_0_* = 20 kW/m^2^. The measurement involves placing the calorimeter directly behind the sample and recording the time (in seconds) it takes the calorimeter temperature to rise by 12 and 24 °C (t_12_ and t_24_, respectively). The result for the particular material or material system is RHTI_24_, which is numerically equal to the arithmetic mean of the three t_24_ values. In addition, it is possible to determine a transmission factor TF, which is the quotient of the density of the heat flux penetrating through the material and the heat flux incident on the material, expressed in percent [54].

In order to assess the resistance of the developed smart textile materials with SMA elements to radiant heat, laboratory tests were carried out according to a specially developed methodology of testing based on the assumptions of EN ISO 6942: 2002 [54]. The traditional test method was modified due to the change in thickness of the smart textile materials during the exposure to radiant heat [10]. The new research methodology allows the testing of smart textile materials characterized by a variable geometry under the influence of thermal radiation and involves maintaining the front surface of the sample on an optical curtain line, in a location where the density of incident heat flux Q_o_ is strictly defined (20 kW/m^2^). Therefore, the results from the tests performed according to the basic and developed method could be compared.

**Figure 5:**
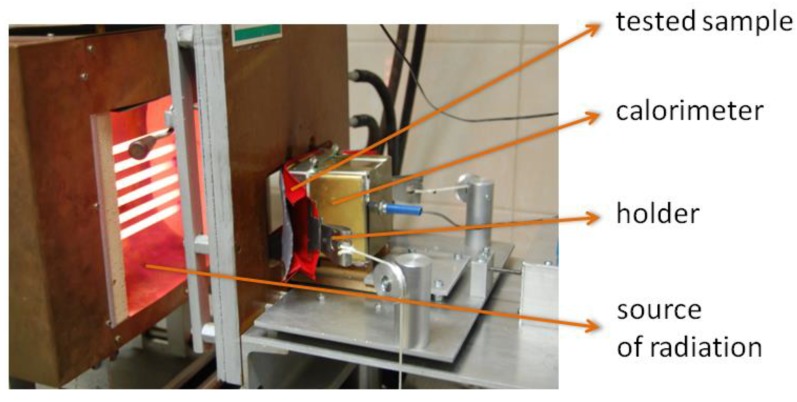
Stand for determining the resistance of materials to radiant heat adapted to the testing of materials with SMA elements.

The special automatic calorimeter motion system enables the calorimeter to automatically adjust its position by linear movement in the case of dimensional changes of the sample during the exposure to radiant heat (Figure 5). The outer surface of the sample remains in a plane in which it is exposed to thermal radiation, and the calorimeter face stays in contact with the moving part of the smart textile material sample. During the test, the time it takes the calorimeter temperature to increase by 12 and 24 °C and the distance moved by the calorimeter corresponding to the change in the sample thickness are recorded. This methodology enables a dynamic assessment of the protective properties of the smart textile material with SMA elements and an assessment of the thickness change effect resulting from the impact of the thermal stimulus.

Moreover, a special method for mounting the samples was designed that included two holders at each side of the sample—one for the top layer and one for the bottom layer. Such an approach guaranteed that the load required for keeping the sample in a relevant position was not too big to limit the increase of the sample thickness during the test. As a consequence, at both sides of the samples, an opening was created through which some convective heat may be transferred. Nevertheless, the same approach was applied to both samples with SMAs, as well as to reference samples. What is more, in the case of standard multilayer samples, they are also open at the sides.

**Figure 6:**
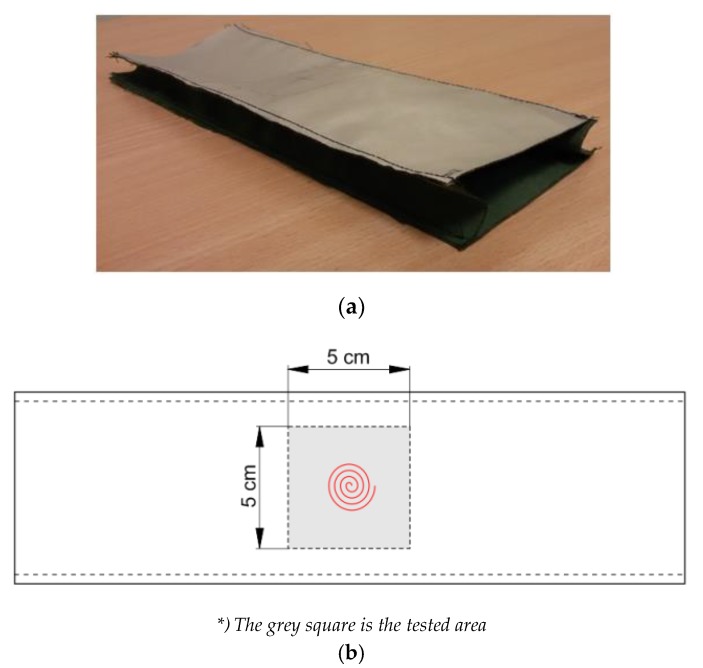
The sample of smart textile materials with SMA elements for the test of resistance to radiant heat: (**a**) a photo and (**b**) a scheme.

In view of the fact that during the exposure of the smart material sample to radiant heat, it expands, creating an open air gap between the outer fabric and the lining, a special method for the preparation of smart textile material for these tests was developed. The samples were sewn along the long sides, creating an additional fold of the lining fabric, thereby reducing the heat exchange with the environment during the test of resistance to radiant heat. A test sample prepared in this way reproduces the layout of materials in clothing, so during the radiant heat resistance test, actual use conditions are simulated. A scheme and photo of this sample are shown in Figure 6.

### 3.3. Tests of Resistance to Molten Iron Splash

The test of resistance to molten iron splash was carried out according to EN ISO 9185:2007 [55]. The test involves pouring a certain amount of molten metal (in this case, iron at a temperature of 1420 °C) onto a sample of material that is placed on a small frame positioned at the angle of 75 ° to the horizontal plane. Directly under the test sample, under its first layer, a special PVC (polyvinyl chloride) film is placed. The film simulates the skin of the wearer of the garment. During the test, the smallest mass of molten metal, which, after pouring onto the sample, causes damage to the PVC film underneath, is determined. The clothing products and/or clothing systems, which are designed to protect against molten iron splash, are classified in accordance with EN ISO 11612:2015 [50] (Table 5).

The highest class of material resistance to molten iron is E3, which is attained when the PVC film under the material is not damaged after pouring >200 g of molten iron onto the material. Due to the fact that the preliminary test results showed that the ASP material with aluminized fabric and SMA elements, tested in accordance with EN ISO 9185:2007 [55], demonstrated resistance to molten iron splashes (440 g) significantly exceeding the E3 level, and at the same time, the aluminized fabric (A) was not damaged by the outpour of 210 g of molten iron (E3), the test method was modified by changing the orientation angle of the fabric during the metal outpour so as to make the test conditions more aggressive. The following changes were introduced to the test method for materials with aluminized fabric:

- The angle of the sample relative to the place of metal discharge was changed to 60° instead of 75°, and
- The time of metal outpour onto the sample was changed to 7.5 s instead of 2.5 s.

The test methodology was modified so as to simulate the situation of potentially longer contact of the fabric with molten, liquid metal. Such a situation may take place while performing activities in conditions of direct proximity to molten metal, such as for example during the operation of a metallurgical furnace.

### 3.4. Statistical Analysis

The obtained results of t_12_, t_24_, and TF were subjected to statistical analysis, which included performing a Shapiro–Wilk test of normality and Levene’s test in order to assess the equality of variances. In the case when those basic assumptions were met, a parametric one-way analysis of variance (ANOVA) test and Tukey post hoc test were carried out in order to indicate statistically significant differences between the analyzed variants. In the case when either the normality of distribution or equality of variances was not met, a non-parametric Kruskal–Wallis test and multiple comparison test were carried out. The significance level 0.05 was adopted. Statistical analysis was carried out using STATISTICA 12 (StatSoft).

Statistical analysis for the change in sample thickness in the test of resistance to ignition was not performed, as it was not possible to compare the obtained results with other variants (change in thickness can be observed only in the case when SMA were applied).

## 4. Results and Discussion

### 4.1. The Results of Resistance to Ignition

The test results of resistance to the ignition of the smart and inactive materials selected for the construction of new protective clothing are demonstrated in Table 6.

In accordance with the requirements of EN ISO 11612:2015 [50], samples of materials for protective clothing should not burn to the top or to the vertical edges, and they should not form holes or flaming or melted debris. The time of consequential burning and glowing should not exceed 2 s. The test results (Table 6) suggest that all test samples met the requirements of the standard. In addition, tests of the active textile system (ASP) exhibited that as a result of the exposure to flames and the shape change of the active elements, the thickness of the ASP textile system increased by more than 40 mm. The actuation of SMA occurred immediately after the application of flame. This means that the active elements applied were properly designed in terms of protection against the heat from ignition because of the additional layer of air between the garment layers.

### 4.2. Test Results of Resistance to Radiant Heat

**Figure 7:**
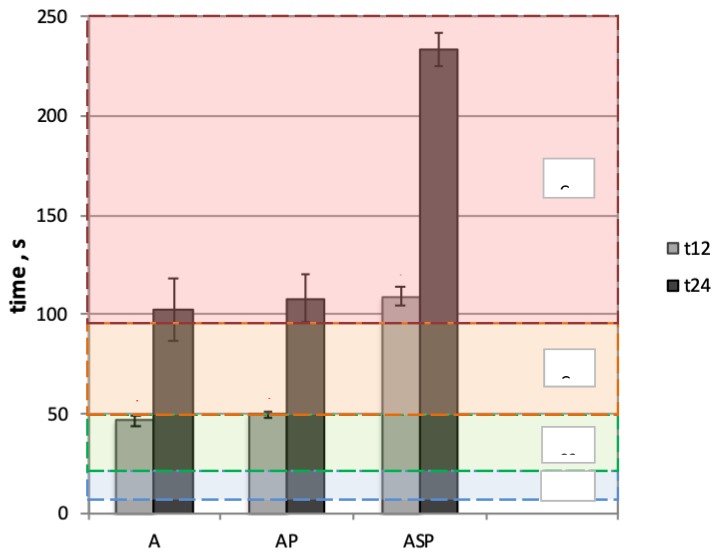
Test result for the time it took the calorimeter temperature to rise by 12 and 24 °C with a heat flux density of 20 kW/m^2^, where * represents a statistically significant difference (*p* < 0.05).

**Figure 8:**
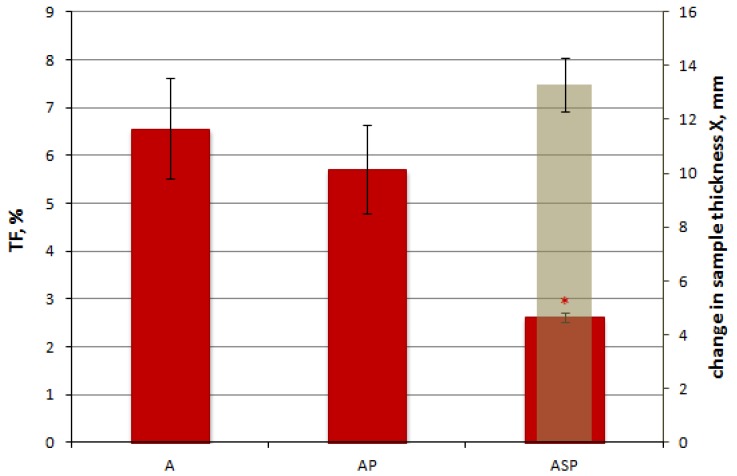
Test results of the heat transmission factor (TF).

The test results of resistance to radiant heat represented by a calorimeter temperature rise of 12 °C (t_12_) and 24 °C (t_24_), as well as the transmission factor (TF) for materials A, AP, and ASP are shown in Figure 7 and Figure 8, respectively. 

On the basis of the obtained results (Figure 7), it can be concluded that adding the lining to the aluminized fabric did not significantly contribute to improvement of the protective properties, and the results obtained for the variant with lining (AP) and the variant with the single layer of fabric (A) are comparable; there is no statistical difference between them. On the other hand, the implementation of active elements in the AP system with aluminized fabric statistically significantly affected the improvement of protective properties compared with both the AP material system and the single outer fabric layer (A). Due to the smart SMA elements, the t_24_ value increased by more than 100%, from 108.1 s for the AP system to 233.6 s for the smart ASP material. These results are in coherence with the studies conducted by He et al. [38], who also analyzed the influence of SMA elements on the resistance to radiant heat of fabric assemblies. For this purpose, the authors used the thermal protective performance (TPP) tester and a heat flux of 12 kW/m^2^. On the basis of the temperature on the internal surface of the thermal liner, they calculated the time t_44_ and t_56_, i.e., the time required to reach the temperature of 44 and 56 °C, respectively. It turned out that due to the presence of SMA elements in the fabric assembly, the value of the t_56_ index increased by 23–81% (depending on the SMA arrangement modes) compared to the control system without SMA elements. Similarly, the study conducted by Congalton [26], who used a conical calorimeter (heat flux of 10 kW/m^2^), showed that the use of an SMA spring in the textile system allowed its protective properties to improve by increasing the time to second degree burn, by even more than 100% (from 38 s for a fabric system without an SMA spring to 78 s for a system with an SMA spring). 

The beneficial effect of implementing SMA elements in the material system is also visible in the value of the transmission factor TF (Figure 8). The value of this index for the ASP variant was only approximately 2.6% and was less than 50% for the AP system. The average value of the increase in thickness of the active material as a result of exposure to the heat impulse in the form of radiant heat was 13.3 mm. 

Moreover, the mass per unit area of the developed material with SMA elements was about 510 g/m^2^, while the mass per unit area of the exemplary material system used in multilayer aluminized clothing providing the same level of protection against radiant heat (C4) was as much as 760 g/m^2^. Therefore, the active material with SMA elements is about 33% lighter than the system commonly used in protective clothing for metallurgists (containing a thick insulating layer instead of SMA elements), but this does not affect the reduction in the level of protection against heat factors. 

### 4.3. Test Results of Resistance to Molten Iron Splash

The test results of resistance to molten iron splash according to the relevant standard and modified method are shown in Table 7 and Table 8 respectively.

The results of the tests conducted according to the relevant standard demonstrated that for both the variant fabric A alone and the variant with lined aluminized fabric (AP), as well as for the system with SMA active elements (ASP), the highest resistance class—E3—was attained.

The results of the tests of resistance to molten iron splash carried out according to the modified test methods indicate that the active ASP material, owing to the application of active SMA elements, is characterized by better protective properties than the AP material without active SMA elements. This is because during the test of the material sample with active SMA elements, no damage to the PVC film was inflicted as a result of the outpouring of 300 g of molten iron, which indicates a very high resistance to molten metal splash. Under similar test conditions for the AP system, the film under the sample was damaged. This means that a significant improvement of protective properties against molten metal was obtained in the case of materials with SMA elements compared to those without SMAs.

Based on the obtained test results (Table 7 and Table 8), it should be noted that the exposure to molten metal splash, in accordance with the presumptions, induced a change in thickness of the active ASP materials at the level of approximately 17 mm. It is noteworthy that the sample thickness change occurred despite the load of molten metal. The presented research results suggest that the implementation of active elements in protective clothing will improve its protective properties against molten metal splashes. 

By analyzing the changes in the thickness of smart material in tests related to the influence of thermal factors, it can be concluded that depending on the type of test, different results were obtained. This is due to the conditions resulting from the method itself and in the case of the test of resistance to molten iron splash, the limiting factor for the expansion of the SMA spring was the weight of molten metal pouring onto the surface of the sample. The largest change in the thickness of samples (i.e., 41 mm) was noted during the test of resistance to ignition, which was mainly due to the close proximity of the flame to the sample. 

## 5. Conclusions

A developed smart material with SMA elements can act as a supplement to currently used materials in clothing protecting against heat, which are usually heavy, multilayered, and give a high level of protection for the whole time of clothing use. The application of smart SMA elements to clothing allowed for the use of much lighter and thinner materials and a beneficial achievement, which is the adaptation of protective properties of clothing to changing working environment conditions. The influence of using SMA elements on the improvement of protective properties of clothing has been confirmed by the results of laboratory tests of the material systems used in it. Tests on resistance to radiant heat showed that the use of active elements in a system with aluminized fabric and lining caused more than a 100% increase in the t_24_ from 108 s for a normal system to 224 s for a system with active SMA elements. As a result of the action of radiant heat flux, the SMA elements expanded and formed an air gap of approximately 13.3 mm between the layers of the system. The beneficial effect of the implementation of smart elements in clothing material on the protective properties of the system obtained in this way was also noticed during the test of resistance to molten iron splash (increase in thickness of 17 mm), as well as resistance to ignition (increase in thickness of 41 mm). Therefore, these results confirm that the smart clothing made of a system with SMA elements can be used, even in conditions of extreme exposure to thermal factors, as it ensures a very good performance in terms of protective properties of the outer layer, as well as additional distance between the outer fabric and the user’s skin when exposed to the thermal factor. 

## Figures and Tables

**Table 1:** The fabrics selected for the smart textile material with SMA elements.

No.	Symbol	Name of Fabric and Raw Material Composition	Mass per Unit Area *, g/m^2^	Thickness ** mm	Designation Acc. to EN ISO 11612:2015 [50]
1	A	Outer fabric: Aluminized woven fabric 50% oxidized poliacrylonitrile, 50% para-aramid	340	0.6	Outer fabric—for protection against a high level of radiant heat. High performance of protection against molten metal splashes (iron)
2	P	Inner fabric: 100% meta-aramid	165	0.4	Lining fabric, flame-retardant

* EN 12127:1997 [51], ** EN ISO 5084:1996 [52].

**Table 2:** Textile materials. A: aluminized woven fabric with para-aramid and oxidized poliacrylonitrile fibers, P: 100% aramid fibers.

Symbol	Structure	Mass per Unit Area, g/m^2^
ASP	outer fabric, A, active elements SMA, S, lining fabric, P	570
AP	outer fabric, A, lining fabric, P	511

**Table 3:** Test methods.

Tested Property	Test Method
Resistance to ignition	EN ISO 15025:2016 [53]
Resistance to radiant heat	EN ISO 6942:2002 [54], according to the modified method
Resistance to large molten metal splash—iron	EN ISO 9185:2007 [55], for aluminized fabrics—modified method

**Table 4:** Performance levels: radiant heat test [50].

Performance Levels	Heat Transfer Factor RHTI_24_, s	
	Min.	Max.
C1	7.0	<20.0
C2	20.0	<50.0
C3	50.0	<95.0
C4	95.0	

**Table 5:** Performance levels: molten iron splash [51].

Performance Levels	Molten Iron Splash, g	
	Min.	Max.
E1	60	<120
E2	120	<200
E3	200	

**Table 6:** Test results of resistance to ignition in accordance with EN ISO 15025:2016 [53].

Test Object	After-Flame Time, s	After-Glow Time, s	Change in Sample Thickness, mm	Comments on the Behavior of the Material during Combustion
A	0	0	-	- the samples did not burn to the top or vertical edge, - no holes were formed, - no flaming or molten debris was found.
P	0	0	-	
ASP	0	0	41.0 ± 2.0	
AP	0	0	-	

**Table 7:** Test results of resistance to molten iron splash in accordance with EN ISO 9185:2007 [55]: the angle of iron outpour onto the sample was 75° and the time of metal outpour onto the sample was 2.5 s.

#	Material	Protective Parameter		Performance Level in Accordance with EN ISO 11612:2015 [50]	Change in Sample Thickness X, mm	Mean of X, mm (SD)
		Mass of Metal Poured, g	Molten Metal Splash Index			
1	ASP	220 220 220	>200	E3	18 15 18	17 (2)
2	AP	220 220 220	>200	E3	-	-
3	A	220 220 220	>200	E3	-	-

SD—standard deviation. **Note:** For protective parameters (“mass of metal poured” and “molten metal splash index”), the mean and standard deviation (SD) were not calculated, because these parameters were obtained by trial-and-error by repeating the procedure of pouring a smaller or larger amount of metal, until the minimum amount of metal that initiated PVC (polyvinyl chloride) film damage was determined.

**Table 8:** Test results of resistance to molten iron splash according to the modified test method: the angle of iron outpour onto the sample was 60° and the time of metal outpour onto the sample was 7.5 s.

#	Material	Protective Parameter		Change in Sample Thickness X, mm	Mean of X, mm (SD)
		Mass of Metal Poured, g	Molten Metal Splash Index		
1	ASP	300 300 300	300	20 17 18	18 (2)
2	AP	300 300 300	The damage of PVC film	-	-

SD—standard deviation. **Note:** For protective parameters (“mass of metal poured” and “molten metal splash index”), the mean and standard deviation (SD) were not calculated, because these parameters were obtained by trial-and-error by repeating the procedure of pouring a smaller or larger amount of metal, until the minimum amount of metal that initiated PVC (polyvinyl chloride) film damage was determined.
